# Patient-centered care in Coronary Heart Disease: what do you want to measure? A systematic review of reviews on patient-reported outcome measures

**DOI:** 10.1007/s11136-022-03260-6

**Published:** 2022-11-09

**Authors:** Yolanda Pardo, Olatz Garin, Cristina Oriol, Víctor Zamora, Aida Ribera, Montserrat Ferrer

**Affiliations:** 1 CIBER Epidemiología y Salud Pública (CIBERESP), Dr. Aiguader 88, 08003 Barcelona, Spain; 2 Health Services Research Group, IMIM (Hospital del Mar Medical Research Institute), Barcelona, Spain; 3 Universitat Autònoma de Barcelona (UAB), Barcelona, Spain; 4 Universitat Pompeu Fabra (UPF), Barcelona, Spain; 5 Unitat de Suport a la Recerca Metropolitana Nord, Institut Universitari d’Investigació en Atenció Primària Jordi Gol (IDIAP Jordi Gol), Mataró, Spain; 6 Cardiovascular Epidemiology and Research Unit, University Hospital and Research Institute Vall d’Hebron (VHIR), Barcelona, Spain

**Keywords:** Patient-reported outcomes, Coronary Heart Disease, Symptoms scale, Health-related quality of life, Functional status

## Abstract

**Background:**

The number of published articles on Patient-Reported Outcomes Measures (PROMs) in Coronary Heart Disease (CHD), a leading cause of disability-adjusted life years lost worldwide, has been growing in the last decades. The aim of this study was to identify all the disease-specific PROMs developed for or used in CHD and summarize their characteristics (regardless of the construct), to facilitate the selection of the most adequate one for each purpose.

**Methods:**

A systematic review of reviews was conducted in MEDLINE, Scopus, and the Cochrane Database of Systematic Reviews. PROQOLID and BiblioPRO libraries were also checked. PROMs were classified by construct and information was extracted from different sources regarding their main characteristics such as aim, number of items, specific dimensions, original language, and metric properties that have been assessed.

**Results:**

After title and abstract screening of 1224 articles, 114 publications were included for full text review. Finally, we identified 56 PROMs: 12 symptoms scales, 3 measuring functional status, 21 measuring Health-Related Quality of Life (HRQL), and 20 focused on other constructs. Three of the symptoms scales were specifically designed for a study (no metric properties evaluated), and only five have been included in a published study in the last decade. Regarding functional status, reliability and validity have been assessed for Duke Activity Index and Seattle Angina Questionnaire, which present multiple language versions. For HRQL, most of the PROMs included physical, emotional, and social domains. Responsiveness has only been evaluated for 10 out the 21 HRQL PROMs identified. Other constructs included psychological aspects, self-efficacy, attitudes, perceptions, threats and expectations about the treatment, knowledge, adjustment, or limitation for work, social support, or self-care.

**Conclusions:**

There is a wide variety of instruments to assess the patients’ perspective in CHD, covering several constructs. This is the first systematic review of specific PROMs for CHD including all constructs. It has practical significance, as it summarizes relevant information that may help clinicians, researchers, and other healthcare stakeholders to choose the most adequate instrument for promoting shared decision making in a trend towards value-based healthcare.

**Supplementary Information:**

The online version contains supplementary material available at 10.1007/s11136-022-03260-6.

## Background

Different initiatives have converged on the importance of the patients’ perspective in the improvement of healthcare. The Institute of Medicine (2001) [1] identified patient-centered care as one of the six domains of high-quality healthcare, wherein patient-centered care supports clinicians in “attending to their patients’ physical and emotional needs and maintaining or improving their quality of life.” The Patient-Centered Outcomes Research Institute [2] emphasizes the goal of “focusing on outcomes that people notice and care about such as survival, function, symptoms, and health-related quality of life.” The American Heart Association (AHA, 2013) [3] states that *Patient-Reported Outcome Measures (PROMs)* implementation in clinical settings has “the potential to support clinical care, evaluate healthcare quality, quantify an important component of procedural appropriateness, identify patients for prognostic discussions and serve as a foundation for shared medical decision making.”

A *patient-reported outcome* has been defined [4] as “a measurement based on a report that comes directly from the patient (i.e., study subject) about the status of a patient’s health condition without amendment or interpretation of the patient’s response by a clinician or anyone else.” PROMS are standardized questionnaires that collect information on health outcomes directly from patients and cover a wide variety of constructs, including symptoms, functional status, and *Health-Related Quality of Life (HRQL)* among others [5]. *HRQL* is one of the constructs most commonly associated with PROMs, and it is a term referring to the health aspects of quality of life, generally considered to reflect the impact of disease and treatment on disability and daily functioning; it has also been considered to reflect the impact of perceived health on an individual's ability to live a fulfilling life [6]. PROMs should ideally undergo psychometric validation to ensure that they accurately reflect the outcomes they purport to cover, and that they are reliable and can assess changes over time.

*Coronary Heart Disease (CHD)* has been the leading cause of disability-adjusted life years lost worldwide since 1990 in people over 50 years old [7]. For patients with CHD, the principal treatment goals are to reduce cardiac events, eradicate angina, and optimize quality of life [8]. In recent decades, the number of published articles reporting the use of PROMs in CHD has been growing, following the global tendency in most chronic pathologies [9]. The proportion of cardiovascular trials evaluating quality of life has increased over time: from 0.34% in 1980 to 3.6% in 1997 [10], to 14% in 2009 [11]; whereas patient-centered outcomes were reported in 29% of cardiac surgery trials performed between 2010 and 2014 [12].

Broadly, PROMs fall into two main categories: condition-specific and generic. The latter measures health concepts that are relevant to a wide range of patient groups, enabling aggregation and comparisons across varied conditions and settings. Condition-specific PROMs capture elements of health relevant to a particular group of patients [13], which in the case of the present review will be CHD patients. Given the growing interest in these instruments, there are several reviews of the main characteristics of some of the most widely used PROMs for CHD, but none have examined all the available instruments [12, 14–20].

These reviews have focused on heart disease in general, on specific heart conditions or on particular constructs (such as symptoms or HRQL) [16]. A review of instruments for patients undergoing elective coronary revascularization identified 26 cardiovascular-specific PROMs [18]. Reviews for specific constructs in CHD have been mainly centered on HRQL [10–14], showing that mostly generic instruments, such as the SF-36 or the EQ-5D, have been used in this pathology. A review centered on symptom scales [20] found 36 different instruments (both generic and specific measures) for all types of cardiovascular populations, including 15 for CHD and angina pectoris. A recent scoping review of validated PROMs including more than one domain, either developed or specifically modified for patients with heart disease, found 9 specific instruments for ischemic heart disease and 5 more applied to all types of heart disease [18].

These numerous reviews result in a kind of puzzle, with partial information and overlaps which make it difficult to obtain the global picture. Therefore, the aim of this study was to identify all the disease-specific PROMs (regardless of the construct) developed for or used in CHD and to describe their main characteristics by conducting a systematic review of reviews. The review protocol is registered in the International Prospective Register of Systematic Reviews database (PROSPERO CRD42021248504).

## Methods

A systematic review of reviews was conducted following the same process as systematic reviews of primary research, but where the units of analysis were reviews rather than individual studies. Following Cochrane recommendations of selecting a minimum of two databases, searches were performed in three electronic databases: MEDLINE, Scopus, and the Cochrane Database of Systematic Reviews. We selected MEDLINE because most of the publications on PROMs are in the field of medical journals and Scopus because it is multidisciplinary and covers other areas. Additionally, we searched in two specialized libraries: PROQOLID (Patient-Reported Outcomes and Quality of Life Instruments Database), and BiblioPRO (a virtual library of PROMs in Spanish). As recommended by the Cochrane Database of Systematic Reviews, the reference lists of relevant studies were also checked to find other potential eligible studies [21].

We applied a comprehensive search strategy developed by experts in Patient-Reported Outcomes and experts in Systematic Reviews in cardiology, seeking to identify published reviews mentioning disease-specific PROMs for CHD populations. For the electronic databases, we used Boolean search methods to identify relevant papers. The search strategy was limited to “reviews” or “systematic reviews” (see supplementary material).

All the identified review articles were included, since the date of each database's inception until March 2021, regardless of whether they were systematic or not. The search was not restricted to language or timeframe.

The inclusion criteria were (1) systematic review or reviews; and (2) providing information about PROMs specifically designed for CHD or, in the case of being designed for heart diseases in general, having been applied and/or validated in patients with CHD; (3) including any type of construct measured: health-related quality of life, symptoms scales, functional status or activities of daily living, and psychological- or social-related construct; (4) instruments that are domain-specific (such as depression, anxiety, self-efficacy, or social support related to CHD) or treatment-specific (for example, coronary artery bypass grafting—CABS—or percutaneous coronary intervention—PCI).

The following exclusion criteria were applied: (a) review or systematic review reporting on generic PROMs applied to CHD (for example, SF-36 or EQ-5D); and (b) review or systematic review of PROMs specifically developed for children or adolescents (age under 18 years).

Two independent reviewers applied inclusion and exclusion criteria to select titles and abstracts, and a single reviewer performed full text and data extraction. All the PROMs identified were categorized according to the construct measured. In case of discrepancy, consensus was reached with the help of a third expert.

### Data extraction and analysis

A data extraction form was developed to collect the PROMs identified, stratified by construct: HRQL, symptoms, functional status, and others.

For each identified PROM, we located the publication reporting the instrument development and we collected information about their general characteristics: author, year, country of publication, and bibliographic reference of the original development; aim (including the characteristics, conditions, or procedure of the population for which the PROM was developed, e.g., coronary artery bypass grafting, congestive heart failure, or myocardial infarction); number of items and dimensions; and the original language and existence of other versions. To identify the linguistic adaptations available, we searched in the PROQOLID and BiblioPRO libraries or the instrument's website. If alternative versions of a PROM were available (for example short forms or pre- and post-treatment forms), they were considered as part of the original. Additionally, we performed a search in PubMed and Scopus databases using the name of the instrument as a search term to find publications of their use within the last 10 years (from March 2011 to March 2021).

For each instrument, we reviewed if there was available information on the main metric properties in patients with CHD: reliability (including reproducibility or test–retest reliability and internal consistency), validity (content, criterion, or construct validity), and sensitivity to change (responsiveness). For information on the metric properties, we considered information on both the original instrument and the linguistic adaptations.

The review was conducted and has been reported in accordance with the Preferred Reporting Items for Systematic Reviews and Meta-Analyses (PRISMA) guidelines [22].

## Results

**Fig. 1:**
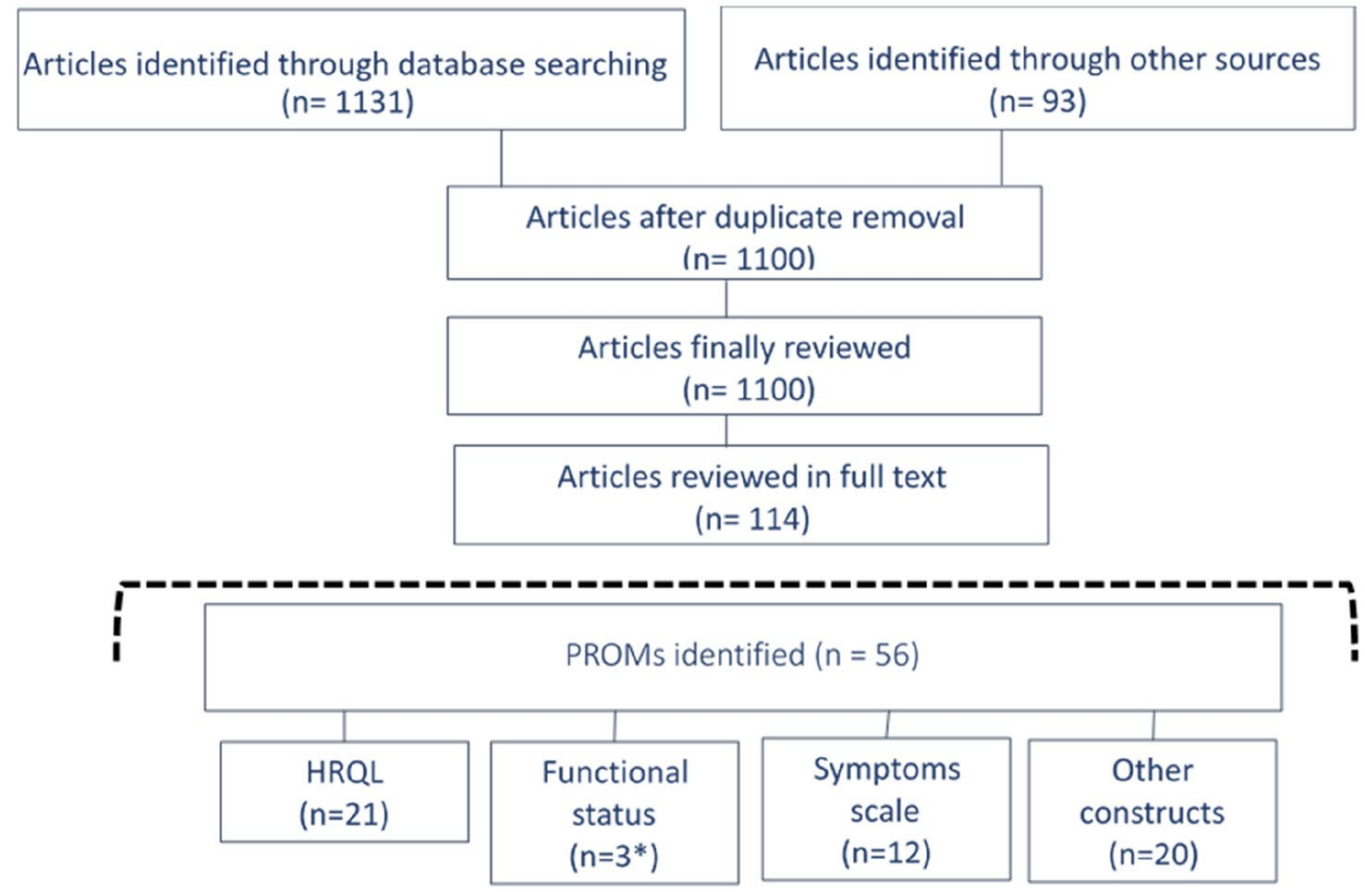
Systematic Review Flow Chart. *One instrument (CLASP) could be included in the “Functional status” or “Symptoms scale” constructs

The search strategy yielded 1131 review articles, and 93 more were added from other sources such as manual reference screening (Fig. 1). After the title and abstract screening, 114 publications were included in the full text review. From these reviews, we identified 56 PROMs specifically developed to be applied in patients with CHD: 21 measuring HRQL (1 of them measuring both symptoms and HRQL, the “Speak from the Heart” instrument), 12 symptoms scales, 3 instruments measuring functional status (1 of them measuring both functional status and symptoms, the Cardiovascular Limitations and Symptoms Profile), and 20 focused on other constructs.

Characteristics of PROMs assessing symptoms and functional status are summarized in Table 1, ordered by the construct measured (symptoms or functional status) and year of publication. Of the 12 symptoms scales identified, three were specifically designed ad hoc for a particular study without any psychometric validation. Only the following 5 instruments have been included in a published study in the last 10 years: Rose Angina Questionnaire (RAQ), Symptoms of Acute Coronary Syndromes Inventory (SACSI), McSweeney Acute and Prodromal Myocardial Infarction Symptom Survey (MAPMISS), Cardiac Symptom Survey (CSS), and Cardiovascular Limitations and Symptoms Profile (CLASP). For these five instruments there is information at least on their reliability and construct validity. However, no sensitivity to change data has been reported. All five instruments have two or more language versions. The RAQ deserves a special mention for being the most widely used in epidemiological surveys since its development in 1962, and being adopted by the WHO [23]. The most common symptom assessed among these symptoms scales is chest pain, followed by dyspnea or fatigue.

**Table 1:** Characteristics of PROMs that assess symptoms and functional status

	Publication: Author, year [reference]	Instrument	Acronym	Aim	Number of items	Dimensions	Original language and adaptations	Studies published last 10 years	Reliability	Validity	Responsiveness
1	Rose GA, 1962 [28]	Rose Angina Questionnaire^b^	RAQ	to standardize the identification of angina effort, pain of possible infarction, and intermittent claudication	7		English (UK) + unspecified No. of language versions	Yes	Internal consistency Reproducibility	Criterion validity Construct validity	
2	Herlitz J., 1988 [100]	Cardiac follow-up questionnaire^a^	-	to assess rehospitalization, bypass surgery, smoking habits, working situation, symptoms of chest pain and dyspnea, and medication 5 years after reporting chest pain	?		Swedish	No			
3	Keresztes P, 1993 [30]	Symptoms scale^a^	SS	“to assess level of angina, shortness of breath, fatigue, and the extent to which the symptoms interfere with overall functional ability”	4	Angina Shortness of breath Fatigue	English (USA)	No	Internal consistency	Construct validity	
4	Artinian NT, 1993 [31]	Symptoms Inventory^a^	-	“to measure cardiac surgery-specific recovery”	20		English (UK)	No	Internal consistency		
5	Plach SK, 2001 [32]	Cardiac Symptom Scale^ac^	CSS	“to measure the frequency of physical symptoms that may occur following a cardiac procedure such as angioplasty or heart surgery”	8		English (UK)	No	Internal consistency	Content validity Construct validity	
6	Schroeder S, 2001 [101]	Schroeder questionnaire^a^	-	“to assess clinical long-term the outcome (in patients treated with percutaneous transluminal coronary angioplasty) and it focused on the patient’s medical history as well as clinical status (self-rated health, angina pectoris)”	?		German	No			
7	DeVon HA, 2003 [34]	Symptoms of Acute Coronary Syndromes Inventory	SACSI	“to describe the type (including severity), location and quality of symptoms for unstable angina”	50	Type of symptoms Pain or discomfort location Quality of pain or discomfort	English (USA) + 1 language	Yes	Internal consistency Reproducibility	Content validity Construct validity	
8	McSweeney JC, 2004 [35]	McSweeney Acute and Prodromal Myocardial Infarction Symptom Survey	MAPMISS	“to develop an instrument validly, describing women's prodromal and acute symptoms of myocardial infarction”	67	Acute, prodromal symptom, and demographic and risk factor	English (USA) + 1 language	Yes	Reproducibility	Content validity Criterion validity	
9	Miller KH, 2004 [36]	Cardiac surgery symptom inventory^a^	CSSI	“to assess symptoms commonly experienced by patients with heart disease prior to surgery, and symptoms often reported after coronary artery bypass surgery”	26	Pre- and post-coronary artery bypass surgery symptoms	English (USA)	No	Internal consistency	Content validity	
10	LaPier TK, 2006 [37]	Heart surgery symptom inventory	HSSI^d^	“to assess the impact of disease-specific symptoms in patients following coronary artery bypass surgery”	76	General, cardiac, trunk, lower extremity, and upper extremity symptoms	English (USA)	No	Internal consistency Reproducibility	Content validity Construct validity	
11	Nieveen JL, 2008 [38]	Cardiac symptom survey (CSS) ^a^	CSS	“to examine symptoms and evaluate symptom management in patients who have undergone coronary artery bypass grafting”	10		English (USA) + 1 language	Yes	Internal consistency Reproducibility	Construct validity	
12	Gilead Sciences, 2012 [39]	Speak From The Heart Chronic Angina Checklist		“to allow patients to share with their healthcare provider how angina is affecting their quality of life by logging information about each angina episode”	7		English (USA)	No			
13^e^	Lewin RJ, 2002 [40]	Cardiovascular limitations and symptoms profile	CLASP	“to capture symptoms and functioning in patients with heart disease, specifically ischemic heart disease and/or congestive heart failure!	37	Symptom subscales (angina, shortness of breath, ankle swelling, and tiredness) Functional limitations (mobility, social life and leisure activities, activities within the home, concerns/worries, and sex)	English (UK) + 2 languages	Yes	Internal consistency Reproducibility	Content validity Criterion validity Construct validity	Yes
14^f^	Hlatky MA, 1989 [41]	Duke Activity Status Index^b^	DASI	“to measure functional capacity and aspects of quality of life”	12		English (USA) + 4 languages	Yes	Internal consistency Reproducibility	Content validity Criterion validity Construct validity	Yes
15 ^f^	Spertus JA, 1995 [42]	Seattle Angina Questionnaire (estándar o short form) ^b^	SAQ, SAQ-SF	“to quantify the physical and emotional effects of coronary artery disease”	19	Physical limitation Angina stability Angina frequency Treatment satisfaction Disease perception Quality of life	English (USA) + 54 languages	Yes	Internal consistency Reproducibility	Content validity Criterion validity Construct validity	Yes

^a^Developed for the study

^b^Different versions available

^c^Instrument only applied to women

^d^In this instrument all patients completed the first 3 sections (general, cardiac, and trunk), and those who had undergone saphenous vein or radial artery harvesting also completed the lower extremity or upper extremity sections, respectively

^e^This instrument is considered a symptom and functional status scale

^f^These instruments are considered functional status scales

? Number of items not reported

Only two of the identified instruments focus on functional status: Seattle Angina Questionnaire (SAQ) and Duke Activity Status Index (DASI). Both were specifically developed for CHD patients, although DASI has nowadays been extended to other conditions. The SAQ, a 19-item questionnaire developed for assessing angina pectoris, has been translated into 54 languages, and a short form is available with seven items. The DASI has 12 items and is available in several languages. Both instruments have evidence on their metric properties: reliability, validity, and responsiveness (Table 1).

Table 2 shows the characteristics of PROMs assessing HRQL, ordered by year of publication between 1988 and 2019. The first ones were developed in Scandinavia for patients with CHD, and most were originally developed in English (11 out of 21 are from UK, USA, Canada, or Australia). Four out of these 21 PROMs were specifically designed for measuring HRQL of patients receiving surgical, percutaneous revascularization or antiarrhythmic medications: Questionnaire for coronary artery bypass grafting, Coronary Revascularization Outcomes Questionnaire (CROQ), Monash University Cardiac Patient-reported Outcome Measure (MC-PROM), and Cardiac Arrhythmia Suppression Trial (CAST). The number of items included in HRQL instruments varies widely, from 14 (HeartQoL), to 70 (Ferrans and Powers QLI-cardiac version). Although the Chronic Heart Failure (CHF) questionnaire was originally designed for heart failure and there are many country-specific versions, we have only included the version that was modified to evaluate HRQL in CHD [24].

**Table 2:** Characteristics of PROMs that assess HRQL

	Publication: Author, year [reference]	Instrument	Acronym	Aim	Structure (number of items and dimensions)	Original language and adaptations	Studies published last 10 years	Reliability	Validity	Responsiveness
1	Wiklund I., 1988 [45]	Angina Pectoris Quality of Life	APQLQ	“to assess disease-specific problems and cardiac symptoms in angina pectoris”	22 items (4 dimensions)	Swedish (Sweden) + 8 languages	No	Internal consistency	Content validity Construct validity	
2	Nissinnen A., 1991 [47]	Angina Impact Questionnaire	AIQ	“to supplement the angina-related information of APQLQ and the Psychological General Wellbeing index”	17 items (4 dimensions)	Finnish (Finland)	No	Internal consistency		
3	Wilson A., 1991 [46]	Summary Index for the Assessment of Quality of Life in Angina Pectoris	Summary Index	“a summary measure for quantifying relevant aspects of quality of life in angina pectoris”	51 items (7 dimensions)	Swedish (Sweden)	No	Internal consistency Reproducibility	Content validity Construct validity	Yes
4	Caine N., 1991 [49]	Questionnaire for coronary artery bypass grafting		“to measure specific HRQL”	No. of items not reported (5 dimensions)	English (UK)	No		Construct validity	
5	Wiklund I., 1992 [50]	Cardiac Arrhythmia Suppression Trial (CAST)	CAST	“to assess aspects of quality of life in the Cardiac Arrhythmia Suppression Trial in patients following an acute myocardial infarction”	21 items (8 dimensions)	English (USA and Canada) and Swedish	No	Internal consistency	Construct validity	
6	Bliley AV., 1993 [24]	Ferrans and Powers QLI-cardiac version	QLI- cardiac version	“to measure quality of life in terms of satisfaction with life”	70 items (4 dimensions)	English (USA) + 10 languages	Yes	Internal consistency Reproducibility	Content validity Criterion validity Construct validity	Yes
7	Velasco JA., 1993[51]	Cuestionario de Calidad de Vida de Velasco-del Barrio	CCVPPI	“to assess HRQL in post-infarction patients”	44 items (9 dimensions)	Spanish (Spain) + 1 language	Yes	Internal consistency Reproducibility	Construct validity	
8	Lim LL-Y., 1993 (QLMI) [53], Valentí L., 1996 (MacNew) [52]	MacNew QLMI	MacNew	“to evaluate the impact of treatment for patients with myocardial infarction, angina pectoris, and heart failure”	27 items (3 dimensions)	English (Canada, Australia) + 82 languages	Yes	Internal consistency Reproducibility	Content validity Criterion validity Construct validity	Yes
9	Rukholm E., 1994 [54]	Cardiac Quality of Life Index	CQLI	“to assess quality of life in cardiac patients”	20 items (5 dimensions)	English (Canada)	No	Internal consistency Reproducibility	Content validity Construct validity	
10	Avís, NE., 1996 [55]	Multidimensional Index of Life Quality	MILQ	“a multidimensional measure of health-related quality of life appropriate for patients with cardiovascular disease”	35 items (9 dimensions)	English (USA) + 1 language	Yes	Internal consistency Reproducibility	Content validity Criterion validity Construct validity	
11	Wahrborg, P., 1996 [56]	Cardiac Health Profile-coronary artery disease	CHP-cad	“to assess HRQL in patients with cardiovascular disease”	19 items (3 parts)	Swedish (Sweden) + 13 languages	Yes	Internal consistency Reproducibility	Construct validity	Yes
12	GISSI-Nursing, 1997 [57]	GISII-nursing	GISII-nursing	“to evaluate HRQL on acute myocardial infarction patients”	29 items (9 dimensions)	Italian (Italy)	No		Content validity	
13	Wolinsky FD., 1998 [58]	Chronic heart failure-modified for CHD^a^	CHF-modified	“a disease-specific health status measure adapted for CHD where chest pain is a dominant symptom”	16 items (4 dimensions)	English (Canada)	No	Internal consistency	Construct validity	
14	Martin AL., 1999 [59]	Utility Based Quality of life-Heart questionnaire	UBQ-H	“to assess utility-based quality of life on cardiovascular patients”	32 items (4 dimensions)	English (Australia)	Yes	Internal consistency Reproducibility	Content validity Criterion validity Construct validity	Yes
15	Buchner DA., 2001 [60]	ITG Health-Related Quality-of-Life long and short-forms Measure	ITG-HRQL-SF	“to develop a questionnaire brief enough for monitoring patients with CAD in everyday clinical practice”	24 and 13 items (4 dimensions)	English (USA)	No	Internal consistency	Content validity Construct validity	
16	Thompson DR., 2002 [61]	Myocardial Infarction Dimensional Assessment Scale -35	MIDAS-35	“to develop a short measure of health status for individuals with acute myocardial infarction”	35 items (7 dimensions)	English (UK) + 6 languages	Yes	Internal consistency Reproducibility	Content validity Criterion validity Construct validity	Yes
17	Schroter S., 2004, 2017 [33]	Coronary revascularization outcomes questionnaire	CROQ	“to evaluate health status in patients undergoing coronary artery bypass grafting and percutaneous transluminal coronary angioplasty”	33 items pre- (4 dimensions) and 52 post-coronary artery bypass grafting and 47 items post-percutaneous transluminal coronary angioplasty	English (UK) + 4 languages	Yes	Internal consistency Reproducibility	Content validity Construct validity	Yes
18	Oldridge N., 2014 [62, 66]	HeartQol^b^	HeartQol	“to assess CHD-specific HRQL for making between-diagnosis comparisons following interventions that are routinely used in more than one CHD diagnosis”	14 items (2 dimensions)	Initially developed in 15 languages* + 16 languages	Yes	Internal consistency Reproducibility	Content validity Construct validity	Yes
19	Wan CH., 2014 [64]	Quality of Life Instruments for Chronic Diseases-Coronary Heart Disease	QLICD-CHD	“to assess symptoms, side effects and special mental health of CHD”	16 items (3 dimensions)	Chinese (China)	Yes	Internal consistency Reproducibility	Content validity Construct validity	Yes
20	Chuanmeng Z., 2018 [37]^c^	Patient-reported outcomes instruments system for chronic diseases-Coronary Heart Disease	PROISCD-CHD	“an evaluation tool for China's patient reported outcome with CHD”	30 (general module) + 15 (specific module)	Chinese (China)	Yes		Content validity Construct validity	Yes
21	Soh SE., 2019 [65]	Monash University cardiac patient-reported outcome measure	MC-PROM	“to assess symptoms and feelings following percutaneous intervention”	5 items	English (Australia)	Yes	Internal consistency	Content validity Construct validity	

^a^This instrument was initially developed for Heart Failure, although it was modified for one study to be applied to CHD

^b^HearQol was initially developed in Danish, Dutch, English (Australia, Canada, Ireland, United Kingdom, the United States of America), French, Flemish, German (Austria, Germany, and Switzerland), Hungarian, Italian, Norwegian, Polish, Portuguese, Russian, Spanish (Cuba and Spain), Swedish, and Ukrainian

^c^Only abstract available

Among the questionnaires identified for measuring HRQL in CHD patients, the most used worldwide are Angina Pectoris Quality of Life Questionnaire (APQLQ), QLI-cardiac version, the MacNew, Cardiac Health Profile (CHP), Myocardial Infarction Dimensional Assessment Scale -35 (MIDAS-35), CROQ, and HeartQoL. They all have several linguistic versions and have appeared in published studies in the last ten years (except for the APQLQ). In addition, information on reliability, validity, and responsiveness has been reported for all of them.

The APQLQ was developed in 1988 in Sweden to assess the impact of angina pectoris on patients’ quality of life and has 9 other language versions. The QLI-cardiac version, also known as Ferrans and Powers questionnaire (1993) [24], was developed to measure quality of life in terms of satisfaction with life in patients with heart diseases and has more than 10 country versions. The MacNew was also developed in the early 1990s, initially for myocardial infarction under the name of Quality of Life after Myocardial Infarction (QLMI) and later expanded to evaluate the impact of treatment for patients with myocardial infarction, angina pectoris, and heart failure, and has been adapted to more than 80 languages. The CHP was developed in 1996 with the main aim to assess HRQL in patients with cardiovascular disease and has a specific version for CHD. It has been adapted into 13 languages and is nowadays still applied. The MIDAS-35 was developed approximately one decade later (2002), to be a short measure of health status for individuals with acute myocardial infarction. It was originally created for UK patients, and nowadays there are 7 language versions. The CROQ was specifically designed to evaluate health status in patients undergoing coronary artery disease grafting and percutaneous transluminal coronary angioplasty, and presents different versions according to the moment of administration (pre- or post-intervention) and the type of intervention (coronary artery disease grafting or percutaneous transluminal coronary angioplasty). The HeartQoL (2014) was developed simultaneously in 15 countries and has subsequently been translated into another 16 languages. The original aim was to assess CHD-specific HRQL for making between-diagnosis comparisons following interventions that are routinely used in more than one CHD diagnosis.

Regarding the content of the dimensions, all instruments cover physical and emotional domains, and most of them also include a social dimension (Table 3). Nine of these questionnaires have a specific domain related to disease symptoms, treatments, or their side effects: APQLQ, Angina Impact Questionnaire (AIQ), Questionnaire for coronary artery bypass grafting, CAST, Cardiac Quality of Life Index (CQLI), MIDAS-35, CROQ, Quality of Life Instruments for Chronic Diseases-Coronary Heart Disease (QLICD-CHD), and MC-PROM. Moreover, 13 out of the 21 instruments have a domain covering specific issues such as sleep problems in the APQLQ and Cuestionario de Calidad de Vida de Velasco-del Barrio (CCVPPI), financial aspects in the Questionnaire for coronary artery bypass grafting and Multidimensional Index of Life Quality (MILQ), alertness in the Summary index and CCVPPI, or occupational aspects in the Questionnaire for coronary artery bypass grafting, CAST or MILQ.

**Table 3:** Dimensions measured by HRQL scales

	Instrument	Physical dimension	Emotional or psychological dimension	Social dimension	Other	Disease-related issues
1	APQLQ	Physical limitations (6)	Emotions (5) Life satisfaction (5)			Symptoms (6)
2	AIQ^a^	Physical activities Sleep disorder	Self-control			Impact of disease
3	Summary Index	Physical exertion (9)	Vitality (5) Self-control (5) Emotional function (18) Alertness (4)	Impact of angina in daily life (9)		
4	Questionnaire for coronary artery bypass grafting^a^			Home, leisure, and social and sexual activities	Working life Financial aspects Overall quality of life	Symptoms
5	CAST	Physical functioning (1)	Mental health (1)	Social functioning (3) Perceived social support (1) Social integration (3)	Life events (1) Work status (9) Life satisfaction (1)	Symptoms (1)
6	QLI-IV cardiac version	Health and functioning (15)	Psychological and spiritual status (7)	Social and economic aspects (8) Family and social relationships (5)		
7	CCVPPI	Health (8) Mobility (6) Sleep and rest (3)	Emotional behavior (3) Concerns to the future (3) Alertness (3)	Social relationships (8) Communication (4) Work and leisure time (6)		
8	Macnew^b^	Physical scale (14)	Emotional scale (14)	Social scale (13)		
9	CQLI	Physical wellbeing (4)	Psychosocial wellbeing (8)^c^ Worry (3)		Nutrition (2)	Symptoms (2)
10	MILQ	Physical health (4) Physical functioning (4)	Mental health (4) Cognitive functioning (4)	Social functioning (4) Intimacy (4)	Financial status (4) Relationship with health professionals (4) Productivity (3)	
11	CHP-cad^ad^	Somatic functioning (3)	Emotional functioning (6)	Social functioning (3) Conative functioning (5)	Control over his/her situation (1)	
12	GISII-nursing	Physical limitations (4) Pain (2)	Emotional limitations (3) Mental health (5) Vitality (4)	Social activity (3)	Functional status (6) Quality of life (1)* General health (1)*	
13	CHF-modified	Fatigue (4)	Emotional (7)			Dyspnea (5)
14	UBQ-H	Physical ability (4)	Psychological distress (16) Self-care (4)	Social-usual activities (5)		
15	ITG-HRQL-SF	Extent of chest pain (4, 3)^d^ Physical functioning (7. 3)^d^	Functioning and wellbeing (7, 3)^d^	Social functioning (6, 4)^d^		
16	MIDAS-35	Physical activity (12)	Insecurity (9) Emotional reaction (4) Dependency (3)		Diet (3)	Concerns over medication (2) Side effects (2)
17	CROQ	Physical functioning (8)	Psychological functioning (14) Cognitive functioning (3)		Satisfaction (6)^e^	Symptoms (7) Adverse effects (11 Coronary Artery Bypass Grafting, 6 Percutaneous Transluminal Coronary Angioplasty)^e^
18	HeartQol	Physical scale (10)	Emotional scale (4)			
19	QLICD-CHD		Effect on mental health and daily life (9)			Symptom (6) Effect of medicine (1)
20	PROISCD-CHD^a^	Physical health	Mental health Beliers	Social health	Beliefs	
21	MC-PROM^f^					Overall score (5)

The number of items in each dimension is in parentheses

^a^Information about the number of items in each dimension is not reported

^b^Some items of this instrument score in more than one dimension

^c^This dimension could be in the psychological-emotional or in the social domains

^d^Number of items for the long and short forms

^e^These dimensions are only in the post-revascularizations versions of the instrument

Supplementary Table 4 shows 20 specific PROMs for CHD that measured some construct other than symptoms, functional status or HRQL, for example, psychological and behavioral aspects such as depression, anxiety, anger, or distress (measured by 5 instruments), self-efficacy (4 instruments), attitudes, perceptions, threats, and expectations about the treatment (3 instruments), knowledge (2 instruments), adjustment or limitation to work (2 instruments), social support (2 instruments), or self-care (1 instrument). Most of these instruments were developed in the last 20 years and have been used recently, with at least one publication in the last 10 years. Those with the most language versions developed are the CDS and the SC-CHDI, which are available in 9 and 11 languages, respectively. Regarding psychometric properties, for the majority of these instruments only internal consistency and construct or content validity have been assessed, being CDS the exception as its reproducibility and responsiveness have also been assessed.

Deserving a special mention is the RehaCAT-Cardio project for the development and validation of a computer adaptive test (CAT) for cardiac patients undergoing rehabilitation, which has designed different item banks and scales for the assessment of several constructs, such as activities of daily living, anxiety, treatment motivation, and work capacity.

## Discussion

Facilitating the selection of the most appropriate PROM for a specific aim is the first step needed to increase patient-centered approaches in CHD, an area where only around 29% of trials report using these measures [12]. The most important characteristic to be considered in this decision process should be what is the construct of interest for the study, program, or initiative implemented.

Reviews of PROMs for CHD have been primarily focused on instruments measuring HRQL or symptoms [12, 14–17, 20]. Through a systematic review we have identified 56 PROMs to be applied to patients with CHD, covering different constructs: HRQL, symptoms, functional status, or several psychological or behavioral aspects.

A few reviews have included various constructs, but they were not exclusively centered on CHD. There is a scoping review of all existing disease-specific PROMs for patients with heart disease, including heart diseases in general, ischemic heart disease, heart failure, arrhythmia, valve disease, and/or grown-up congenital heart disease [19], which only identified 9 instruments for ischemic heart disease and 5 for heart disease in general. The low number of PROMs identified by this scoping review was probably due to the exclusion criteria of PROMs measuring single symptoms or domains, because its purpose was mapping the items contained in WHO’s International Classification of Functioning, Disability, and Health (ICF). A systematic review of cardiovascular-specific PROMs identified 26 instruments for patients undergoing elective coronary revascularization, although specific PROMs applied to other procedures were not included [18].

Symptoms and functional status were also commonly included in the CHD studies, and 15 PROMs have been developed. A literature review of PROMs assessing symptoms for different cardiovascular diseases found 14 symptoms instruments for various acute coronary syndromes and 10 specifically for patients with angina [20].

Typically, the quantification method of symptoms and functioning in CHD has been the Canadian Cardiovascular Society (CCS) classification system, which is determined by the clinician rather than the patient [25]. Almost 40 years of research have documented substantial limitations in the CCS classification system [26, 27]: the data collectively suggest a need for more consistent, systematic, and accurate means to quantify the frequency and burden of angina from the patient’s perspective. In this sense, 12 symptoms [28–39] and 3 functional status instruments [40–42] have been developed, but only 5 symptoms and 2 functional status scales have been used in the last 10 years. This is probably the consequence of some of them having been established as “gold standards” and used consistently due to the accumulated evidence of well-established validity, reproducibility, prognostic importance, and sensitivity to clinical change [25]. This would be the case of the RAQ for symptom recognition or the DASI and the SAQ for functional status assessment [9].

The RAQ was constructed in the 1960s for assessing the population burden of angina, and positive screening in RAQ predicts myocardial infarction and cardiovascular disease [43]. The DASI, although initially developed for cardiac patients to assess usual physical activities and cardiopulmonary fitness, has been shown to be useful for non-cardiac disease to improve the identification of patients at an elevated risk for myocardial infarction [44]. The SAQ was endorsed as a performance measure by the AMA/ACC/AHA Physicians’ Consortium for Performance Improvement in the 1990s, and it has been more extensively applied in clinical trials than adopted in clinical practice [27]. The International Consortium for Health Outcomes Measurement (ICHOM), an organization with the mission to unlock the potential of value-based healthcare, recommends the short version of the SAQ in the CHD standard sets of outcome measures.

Instruments focused on HRQL are the largest group with 21 PROMs [24, 33, 45–66], making it difficult to select the most appropriate instrument, yet this is one of the most interesting constructs for clinicians and researchers. HRQL instruments specific for patients with CHD usually include a physical and an emotional domain, quite frequently also a social domain, and the most recent ones add dimensions for measuring self-care, dependency, or satisfaction. Considering the most frequently used instruments in the last 10 years (APQLQ, QLI-cardiac version, MacNew, MIDAS-35, CROQ, and HeartQol), differences among the number of items (22 to 70) and domains are important. Half of them were developed with the aim of evaluating the impact of treatments (MacNew, CROQ, and HeartQol), allowing for comparisons among interventions, a relevant aspect for shared decision making.

In recent decades, there has been a growing interest in patient-centered approaches that open the scope to other constructs like self-care, attitudes, or psychological aspects specifically related to the disease. Our review has found 20 scales [67–91] to measure mood symptoms, perception, expectation regarding the disease (beliefs, attitudes, risk perception, or knowledge) or coping strategies (self-efficacy, self-care, adjustment, or social support). CHD often involves mood symptoms of distress, anxiety, or depression, which, in turn, are risk factors for CHD [92, 93]. However, it should be noted that information on their metric properties has not been reported yet for many of these instruments, with a considerable gap regarding their sensitivity to change. CDS is the only instrument with evidence on reliability, validity, and responsiveness. In the United States, the proportion of depression in patients with acute myocardial infarction is three times higher than in the general population, and anxiety is almost twice [94, 95]. Aware of these data, the American Heart Association (AHA) recommends routine screening for depression, allowing effective treatment for improving health outcomes [96]. Conversely, the assessment of protective factors such as self-care, self-efficacy, or social support, among other constructs related to coping strategies, could help to promote lifestyle changes in patients and increase the level of compliance with medical recommendations.

In general, there is a tendency to develop short versions of the original instrument or to develop a renewed version. Such is the case of the RAQ, which has multiple modified versions, or the MacNew, which was initially developed in 1993 for myocardial infarction (QLMI) and extended its applicability to other conditions in the current version published in 1996. The SAQ, with an original version of 19 items, has a shorter 7-item version developed to reduce its response burden and to provide a single summary score to facilitate its adoption in clinical care [97].

### Strengths and limitations

This is the first systematic review of specific PROMs applied in CHD patients, including all the constructs measured, and without restrictions regarding language and year of publication. It was registered at PROSPERO and follows the PRISMA guidelines for the systematic review of reviews, a research procedure nowadays recommended to increase transparency and reproducibility and decrease reporting bias.

The results of the present systematic review should be interpreted in the light of their principal limitation. The authors acknowledge that more PROMs might have been identified if other databases had been searched in addition to MEDLINE, Scopus, and the Cochrane Database. However, additional manual screening of reference lists from relevant articles and targeted searches in PROQOLID and BiblioPRO still yielded a relatively large number of PROMs.

## Conclusions and practical implications

A wide variety of instruments has been developed to assess patient-reported outcomes in CHD, covering several constructs which may be of special interest. The first step to increase patient-centered approaches in CHD is to identify, classify, and describe the existing PROMs, including the evidence on their metric properties. The second step on the continuum towards value-based healthcare [98] may be the evaluation of PROMs’ implementation in different settings, to report the usability and utility of these measures for patients, carers, and health professionals.

In conclusion, this review has practical significance, as it summarizes relevant information that may help clinicians, researchers, and other healthcare stakeholders to choose the most adequate instrument for incorporating the patients’ perspective and promote a model of shared decision making in a trend towards value-based healthcare [99].

## Supplementary Information

Below is the link to the electronic supplementary material.

Supplementary file1 (DOCX 443 kb)

## Data Availability

Data and material are available from the corresponding author upon reasonable request.

## References

[1] Institute of Medicine (US) Committee on Quality of Health Care in America (2001). Crossing the Quality Chasm: A New Health System for the 21st Century.

[2] Patient-Centered Outcomes Research | PCORI. (n.d.). Retrieved July 30, 2021, from https://www.pcori.org/research-results/about-our-research/patient-centered-outcomes-research

[3] Rumsfeld JS, Alexander KP, Goff DC, Graham MM, Ho PM, Masoudi FA, Moser DK, Roger VL, Slaughter MS, Smolderen KG, Spertus JA, Sullivan MD, Treat-Jacobson D, Zerwic JJ, American Heart Association Council on Quality of Care and Outcomes Research, Council on Cardiovascular and Stroke Nursing, Council on Epidemiology and Prevention, Council on Peripheral Vascular Disease, and Stroke Council (2013). Cardiovascular health: The importance of measuring patient-reported health status a scientific statement from the American Heart Association. Circulation.

[4] U.S. Department of Health and Human Services FDA Center for Drug Evaluation and Research; U.S. Department of Health and Human Services FDA Center for Biologics Evaluation and Research; U.S. Department of Health and Human Services FDA Center for Devices and Radiological Health (2006). Guidance for industry: patient-reported outcome measures: use in medical product development to support labeling claims: draft guidance. Health and Quality of Life Outcomes.

[5] McKenna SP (2011). Measuring patient-reported outcomes: Moving beyond misplaced common sense to hard science. BMC Medicine.

[6] Mayo NE (2015). ISOQOL dictionary of quality of life and health outcomes measurement.

[7] GBD 2019 Diseases and Injuries Collaborators (2020). Global burden of 369 diseases and injuries in 204 countries and territories, 1990–2019: A systematic analysis for the Global Burden of Disease Study 2019. The Lancet (London, England).

[8] Fihn SD, Gardin JM, Abrams J, Berra K, Blankenship JC, Dallas AP, Douglas PS, Foody JM, Gerber TC, Hinderliter AL, King MA, Prager RL, Sabik JF, Shaw LJ, Sikkema JD, Smith CR, Smith SsC, Spertus JA, Williams SW, American College Of Cardiology Foundation (2012). 2012 ACCF/AHA/ACP/AATS/PCNA/SCAI/STS guideline for the diagnosis and management of patients with stable ischemic heart disease: executive summary: a report of the American College of Cardiology Foundation/American Heart Association task force on practice. Circulation.

[9] Garcia RA, Spertus JA (2021). Using patient-reported outcomes to assess healthcare quality: Toward better measurement of patient-centered care in cardiovascular disease. Methodist DeBakey Cardiovascular Journal.

[10] Sanders C, Egger M, Donovan J, Tallon D, Frankel S (1998). Reporting on quality of life in randomised controlled trials: Bibliographic study. BMJ (Clinical Research Edition).

[11] Rahimi K, Malhotra A, Banning AP, Jenkinson C (2010). Outcome selection and role of patient reported outcomes in contemporary cardiovascular trials: Systematic review. BMJ (Clinical Research Edition).

[12] Mark DB (2016). Assessing quality-of-life outcomes in cardiovascular clinical research. Nature Reviews Cardiology.

[13] Churruca K, Pomare C, Ellis LA, Long JC, Henderson SB, Murphy LED, Leahy CJ, Braithwaite J (2021). Patient-reported outcome measures (PROMs): A review of generic and condition-specific measures and a discussion of trends and issues. Health Expectations.

[14] da Silva SA, Passos SRL, Carballo MT, Figueiró MF (2011). Quality of life assessment after acute coronary syndrome: Systematic review. Arquivos Brasileiros de Cardiologia.

[15] Cepeda-Valery B, Cheong AP, Lee A, Yan BP (2011). Measuring health related quality of life in coronary heart disease: The importance of feeling well. International Journal of Cardiology.

[16] Soo Hoo SY, Gallagher R, Elliott D (2014). Systematic review of health-related quality of life in older people following percutaneous coronary intervention. Nursing and Health Sciences.

[17] Khoiriyati, A., Kusnanto, & Kurniawati, N. D. (2020). Selecting instruments to measure quality of life after acute coronary syndrome: A literature review. *International Journal of Psychosocial Rehabilitation*, *24*(7), 7744–7751.

[18] Peeters G, Barker AL, Talevski J, Ackerman I, Ayton DR, Reid C, Evans SM, Stoelwinder JU, McNeil JJ (2018). Do patients have a say? A narrative review of the development of patient-reported outcome measures used in elective procedures for coronary revascularisation. Quality of Life Research.

[19] Algurén B, Coenen M, Malm D, Fridlund B, Mårtensson J, Årestedt K (2020). A scoping review and mapping exercise comparing the content of patient-reported outcome measures (PROMs) across heart disease-specific scales. Journal of Patient-Reported Outcomes.

[20] Zimmerman L, Pozehl B, Vuckovic K, Barnason S, Schulz P, Seo Y, Ryan CJ, Zerwic JJ, DeVon HA (2016). Selecting symptom instruments for cardiovascular populations. Heart and Lung.

[21] Horsley T, Dingwall O, Sampson M (2011). Checking reference lists to find additional studies for systematic reviews. Cochrane Database of Systematic Reviews.

[22] Page, M. J., McKenzie, J. E., Bossuyt, P. M., Boutron, I., Hoffmann, T. C., Mulrow, C. D., Shamseer, L., Tetziaff, J. M., Akl, E. A., Brennan, S. E., Chou, R., Glanville, J., Grimshaw, J. M., Hróbjartsson, A., Lalu, M. M., Li, T., Loder, E. W., Mayo-Wilson, E., McDonal, S., … Moher, D. (2021). The PRISMA 2020 statement: an updated guideline for reporting systematic reviews. *BMJ (Clinical Research Edition)*. 10.1136/bmj.n71 10.1136/bmj.n71 PMC8005924 33782057

[23] Cook DG, Shaper AG, MacFarlane PW (1989). Using the WHO (Rose) angina questionnaire in cardiovascular epidemiology. International Journal of Epidemiology.

[24] Bliley AV, Ferrans CE (1993). Quality of life after coronary angioplasty. Heart and Lung.

[25] Thomas M, Jones PG, Arnold SV, Spertus JA (2021). Interpretation of the Seattle Angina Questionnaire as an outcome measure in clinical trials and clinical Care: A review. JAMA Cardiology.

[26] Beltrame JF, Weekes AJ, Morgan C, Tavella R, Spertus JA (2009). The prevalence of weekly angina among patients with chronic stable angina in primary care practices: The Coronary Artery Disease in General Practice (CADENCE) Study. Archives of Internal Medicine.

[27] Shafiq A, Arnold SV, Gosch K, Kureshi F, Breeding T, Jones PG, Beltrame J, Spertus JA (2016). Patient and physician discordance in reporting symptoms of angina among stable coronary artery disease patients: Insights from the Angina Prevalence and Provider Evaluation of Angina Relief (APPEAR) study. American Heart Journal.

[28] Rose GA (1962). The diagnosis of ischaemic heart pain and intermittent claudication in field surveys. Bulletin of the World Health Organization.

[29] Herlitz J, Hjalmarson R, Karlson BW, Nyberg G, Hjalmarson A, Karlson BW, Nyberg G (1988). Long-term morbidity in patients where the initial suspicion of myocardial infarction was not confirmed. Clinical Cardiology.

[30] Keresztes P, Holm K, Penckofer S, Merritt S (1993). Measurement of functional ability in patients with coronary artery disease. Journal of Nursing Measurement.

[31] Artinian NT, Duggan C, Miller P (1993). Age differences in patient recovery patterns following coronary artery bypass surgery. American Journal of Critical Care.

[32] Plach SK, Heidrich SM (2001). Women’s perceptions of their social roles after heart surgery and coronary angioplasty. Heart and Lung.

[33] Schroter S, Lamping DL (2004). Coronary revascularisation outcome questionnaire (CROQ): Development and validation of a new, patient-based measure of outcome in coronary bypass surgery and angioplasty. Heart.

[34] DeVon HA, Ryan CJ, Ochs AL, Moshe S (2008). Symptoms across the continuum of acute coronary syndromes: Differences between women and men. American Journal of Critical Care.

[35] McSweeney JC, O’Sullivan P, Cody M, Crane PB (2004). Development of the McSweeney acute and prodromal myocardial infarction symptom survey. The Journal of Cardiovascular Nursing.

[36] Miller KH, Grindel CG (2004). Comparison of symptoms of younger and older patients undergoing coronary artery bypass surgery. Clinical Nursing Research.

[37] LaPier TK (2006). Psychometric evaluation of the heart surgery symptom inventory in patients recovering from coronary artery bypass surgery. Journal of Cardiopulmonary Rehabilitation.

[38] Nieveen JL, Zimmerman LM, Barnason SA, Yates BC (2008). Development and content validity testing of the Cardiac Symptom Survey in patients after coronary artery bypass grafting. Heart and Lung.

[39] Gilead Sciences. (2012). The speak from the heart chronic angina checklist. http://www.speakfromtheheart.com/anginaassessment.aspx

[40] Lewin RJP, Thompson DR, Martin CR, Stuckey N, Devlen J, Michaelson S, Maguire P (2002). Validation of the Cardiovascular Limitations and Symptoms Profile (CLASP) in chronic stable angina. Journal of Cardiopulmonary Rehabilitation.

[41] Hlatky MA, Boineau RE, Higginbotham MB, Lee KL, Mark DB, Califf RM, Cobb FR, Pryor DB (1989). A brief self-administered questionnaire to determine functional capacity (the Duke Activity Status Index). The American Journal of Cardiology.

[42] Spertus JA, Winder JA, Dewhurst TA, Deyo RA, Prodzinski J, McDonell M, Fihn SD (1995). Development and evaluation of the Seattle Angina Questionnaire: A new functional status measure for coronary artery disease. Journal of the American College of Cardiology.

[43] Graff-Iversen S, Wilsgaard T, Mathiesen EB, Njølstad I, Løchen M-L (2014). Long-term cardiovascular consequences of Rose angina at age 20–54 years: 29-years’ follow-up of the Tromso Study. Journal of Epidemiology and Community Health.

[44] Wijeysundera, D. N., Beattie, W. S., Hillis, G. S., Abbott, T. E. F., Shulman, M. A., Ackland, G. L., Mazer, C. D., Myles, P. S., Pearse, R. M., Cuthbertson, B. H., Measurement of Exercise Tolerance before Surgery Study Investigators, Myles, P. S., Shulman, M. A., Wallace, S., Farrington, C., Thompson, B., Ellis, M., Borg, B., Kerridge, R K.,… Lifford, R. (2020). Integration of the Duke Activity Status Index into preoperative risk evaluation: a multicentre prospective cohort study. *British Journal of Anaesthesia*, *124*(3), 261–270. 10.1016/j.bja.2019.11.025 10.1016/j.bja.2019.11.025 31864719

[45] Wiklund I (1988). Livskvalitet vid kardiovaskulara sjukdomar. Scandinavian Journal of Behavioral Therapy.

[46] Wilson A, Wiklund I, Lahti T, Wahl M (1991). A summary index for the assessment of quality of life in angina pectoris. Journal of Clinical Epidemiology.

[47] Nissinen A, Wiklund I, Lahti T, Akkila J, Wilson A, Wahl M, Puska P (1991). Anti-anginal therapy and quality of life. A comparison of the effects of transdermal nitroglycerin and long-acting oral nitrates. Journal of Clinical Epidemiology.

[48] Wilson IB, Cleary PD (1995). Linking clinical variables with health-related quality of life. A conceptual model of patient outcomes. JAMA.

[49] Caine N, Harrison SCW, Sharples LD, Wallwork J (1991). Prospective study of quality of life before and after coronary artery bypass grafting. British Medical Journal.

[50] Wiklund I, Gorkin L, Pawitan Y, Schron E, Schoenberger J, Jared LL, Shumaker S (1992). Methods for assessing quality of life in the cardiac arrhythmia suppression trial (CAST). Quality of Life Research.

[51] Velasco JA, del Barrio V, Mestre MV, Penas C, Ridocci F (1993). Validation of a new questionnaire to evaluate the quality of life in patients after myocardial infarction. Revista Espanola de Cardiologia.

[52] Valenti L, Lim L, Heller RF, Knapp J (1996). An improved questionnaire for assessing quality of life after acute myocardial infarction. Quality of Life Research.

[53] Lim LLY, Valenti LA, Knapp JC, Dobson AJ, Plotnikoff R, Higginbotham N, Heller RF (1993). A self-administered quality-of-life questionnaire after acute myocardial infarction. Journal of Clinical Epidemiology.

[54] Rukholm E, McGirr M (1994). A quality-of-life index for clients with ischemic heart disease: Establishing reliability and validity. Rehabilitation Nursing.

[55] Avis NE, Smith KW, Hambleton RK, Feldman HA, Selwyn A, Jacobs A (1996). Development of the multidimensional index of life quality: A quality of life measure for cardiovascular disease. Medical Care.

[56] Währborg P, Emanuelsson H (1996). The cardiac health profile: Content, reliability and validity of a new disease-specific quality of life questionnaire. Coronary Artery Disease.

[57] GISSI-Nursing. (1997). [Evaluation of perception of quality of life and health by patients with myocardial infarction. GISSI-Nursing]. *Giornale Italiano di Cardiologia**, **27*(9), 865–876. 9378191

[58] Wolinsky FD, Wyrwich KW, Nienaber NA, Tierney WM (1998). Generic versus disease-specific health status measures. An example using coronary artery disease and congestive heart failure patients. Evaluation and the Health Professions.

[59] Martin AJ, Glasziou PP, Simes RJ (1999). A cardiovascular extension of the Health Measurement Questionnaire. Journal of Epidemiology and Community Health.

[60] Buchner DA, Graboys TB, Johnson K, Mordin MM, Goodman L, Partsch DS, Goss TF (2001). Development and validation of the ITG Health-Related Quality-of-Life Short-Form measure for use in patients with coronary artery disease. Integrated Therapeutics Group. Clinical Cardiology.

[61] Thompson DR, Jenkinson C, Roebuck A, Lewin RJP, Boyle RM, Chandola T (2002). Development and validation of a short measure of health status for individuals with acute myocardial infarction: The myocardial infarction dimensional assessment scale (MIDAS). Quality of Life Research.

[62] Oldridge, N., Höfer, S., McGee, H., Conroy, R., Doyle, F., Saner, H. (for the HeartQoL Project Investigators). (2014). The HeartQoL: Part II. Validation of a new core health-related quality of life questionnaire for patients with ischemic heart disease. *European Journal of**Preventive Cardiology, 21*(1), 98–106. . Retrieved from https://journals.sagepub.com/doi/abs/10.1177/2047487312450545 10.1177/2047487312450545 22822180

[63] Chuanmeng Z, Zhiwen H, Chonghua W, Zheng Y, Chuanzhi X (2018). Development and responsiveness of the coronary heart disease scale in the patient reported outcomes instruments system for chronic diseases. Chinese Journal of Modern Nurse.

[64] Wan C, Li H, Fan X, Yang R, Pan J, Chen W, Zhao R (2014). Development and validation of the coronary heart disease scale under the system of quality of life instruments for chronic diseases QLICD-CHD: Combinations of classical test theory and Generalizability theory. Health and Quality of Life Outcomes.

[65] Soh S-E, Barker AL, Ayton DR, Ahern S, Morello R, Lefkovits J, Brennan AL, Evans S, Zalcberg JR, Reid CM, McNeil JJ (2019). What matters most to patients following percutaneous coronary interventions? A new patient-reported outcome measure developed using Rasch analysis. PLoS ONE.

[66] Oldridge N, Höfer S, McGee H, Conroy R, Doyle F, Saner H (2014). The HeartQoL: Part I. Development of a new core health-related quality of life questionnaire for patients with ischemic heart disease. European Journal of Preventive Cardiology.

[67] Rumbaugh DM, Knapp RR (1965). Prediction of work potential in heart patients through use of the cardiac adjustment scale. Journal of Consulting Psychology.

[68] Barnason, S. A. (1992). *A comparison of cardiac teaching on learning variables among cardiac surgical patients*. ETD collection for University of Nebraska - Lincoln. AAI9314388.

[69] Barnason S, Zimmerman L, Atwood J, Nieveen J, Schmaderer M (2002). Development of a self-efficacy instrument for coronary artery bypass graft patients. Journal of Nursing Measurement.

[70] Moser DK, Dracup K (1995). Psychosocial recovery from a cardiac event: The influence of perceived control. Heart and Lung.

[71] Riegel B, McKinley S, Moser DK, Meischke H, Doering L, Dracup K (2007). Psychometric evaluation of the Acute Coronary Syndrome (ACS) response index. Research in Nursing and Health.

[72] Hare DL, Davis CR (1996). Cardiac Depression Scale: Validation of a new depression scale for cardiac patients. Journal of Psychosomatic Research.

[73] Bennett SJ, Puntenney PJ, Walker NL, Ashley ND (1996). Development of an instrument to measure threat related to cardiac events. Nursing Research.

[74] Lerner DJ, Amick BC, Malspeis S, Rogers WH, Gomes DR, Salem DN (1998). The angina-related limitations at work questionnaire. Quality of life Research.

[75] Sullivan MD, LaCroix AZ, Russo J, Katon WJ (1998). Self-efficacy and self-reported functional status in coronary heart disease: A six-month prospective study. Psychosomatic Medicine.

[76] The ENRICHD investigators (2000). Enhancing recovery in coronary heart disease patients (ENRICHD): Study design and methods. The ENRICHD investigators. American Heart Journal.

[77] Eifert GH, Thompson RN, Zvolensky MJ, Edwards K, Frazer NL, Haddad JW, Davig J (2000). The Cardiac Anxiety Questionnaire: Development and preliminary validity. Behaviour Research and Therapy.

[78] di Benedetto M, Sheehan M (2014). Evaluation of the Cardiac Depression Visual Analogue Scale in a medical and non-medical sample. Psychology, Health and Medicine.

[79] Young Q-R, Ignaszewski A, Fofonoff D, Kaan A (2007). Brief screen to identify 5 of the most common forms of psychosocial distress in cardiac patients: Validation of the screening tool for psychological distress. Journal of Cardiovascular Nursing.

[80] Haschke A, Abberger B, Wirtz M, Bengel J, Baumeister H (2013). Development of short form questionnaires for the assessment of work capacity in cardiovascular rehabilitation patients. International Journal of Occupational and Environmental Health.

[81] Schmucker A, Abberger B, Boecker M, Baumeister H (2019). Parallel short forms for the assessment of activities of daily living in cardiovascular rehabilitation patients (PADL-cardio): Development and validation. Disability and Rehabilitation.

[82] Abberger B, Haschke A, Krense C, Wirtz M, Bengel J, Baumeister H (2013). The calibrated, unidimensional anxiety item bank for cardiovascular patients provided the basis for anxiety assessment in cardiovascular rehabilitation patients. Journal of Clinical Epidemiology.

[83] Baumeister H, Abberger B, Haschke A, Boecker M, Bengel J, Wirtz M (2013). Development and calibration of an item bank for the assessment of activities of daily living in cardiovascular patients using Rasch analysis. Health and Quality of Life Outcomes.

[84] Abberger B, Haschke A, Wirtz M, Kroehne U, Bengel J, Baumeister H (2013). Development and evaluation of a computer adaptive test to assess anxiety in cardiovascular rehabilitation patients. Archives of Physical Medicine and Rehabilitation.

[85] Abberger B, Haschke A, Tully PJ, Forkmann T, Berger J, Wirtz M, Bengel J, Baumeister H (2017). Development and validation of parallel short forms PaSA-cardio for the assessment of general anxiety in cardiovascular rehabilitation patients using Rasch analysis. Clinical Rehabilitation.

[86] Chan CW (2014). Perceptions of coronary heart disease: The development and psychometric testing of a measurement scale. Psychology, Health and Medicine.

[87] Steca P, Greco A, Cappelletti E, D’Addario M, Monzani D, Pancani L, Ferrari G, Politi A, Gestra R, Malfatto G, Parati G (2015). Cardiovascular management self-efficacy: Psychometric properties of a new scale and its usefulness in a rehabilitation context. Annals of Behavioral Medicine.

[88] Odell A, Bång A, Andréll P, Widell C, Fryklund H, Kallryd A, Tygesen H, Grip L (2017). Patients expectations and fulfilment of expectations before and after treatment for suspected coronary artery disease assessed with a newly developed questionnaire in combination with established health-related quality of life questionnaires. Open Heart.

[89] Vaughan Dickson V, Lee CS, Yehle KS, Mola A, Faulkner KM, Riegel B (2017). Psychometric testing of the Self-Care of Coronary Heart Disease Inventory (SC-CHDI). Research in Nursing and Health.

[90] Jackson A, Rogerson M, le Grande M, Thompson D, Ski C, Alvarenga M, Amerena J, Higgins R, Raciti M, Murphy BM (2020). Protocol for the development and validation of a measure of persistent psychological and emotional distress in cardiac patients: The Cardiac Distress Inventory. British Medical Journal Open.

[91] Haschke A, Abberger B, Müller E, Wirtz M, Bengel J, Baumeister H (2013). Calibration of an item bank for work capacity in cardiological rehabilitation patients. European Journal of Preventive Cardiology.

[92] Carney RM, Freedland KE (2017). Depression and coronary heart disease. Nature Reviews. Cardiology.

[93] Karlsen HR, Matejschek F, Saksvik-Lehouillier I, Langvik E (2021). Anxiety as a risk factor for cardiovascular disease independent of depression: A narrative review of current status and conflicting findings. Health Psychology Open.

[94] Egede LE (2007). Major depression in individuals with chronic medical disorders: Prevalence, correlates and association with health resource utilization, lost productivity and functional disability. General Hospital Psychiatry.

[95] Lee C, Lee SC, Shin YS, Park S, Won KB, Ann SH, Ko EJ (2020). Severity, progress, and related factors of mood disorders in patients with coronary artery disease: A retrospective study. Healthcare.

[96] Lichtman, J. H., Bigger, J. T. J., Blumenthal, J. A., Frasure-Smith, N., Kaufmann, P. G., Lespérance, F., Mark, D. B., Sheps, D. S., Taylor, C. B., Froelicher, E. S., American Heart Association Prevention Committee of the Council on Cardiovascular Nursing; American Heart Association Council on Clinical Cardiology; American Heart Association Council on Epidemiology and Prevention; American Heart Association Interdisciplinary Council on Quality of Care and Outcomes Research; & American Psychiatric Association. (2008). Depression and coronary heart disease: Recommendations for screening, referral, and treatment: A science advisory from the American Heart Association Prevention Committee of the Council on Cardiovascular Nursing, Council on Clinical Cardiology, Council on Epidemiology and Prevention, and Interdisciplinary Council on Quality of Care and Outcomes Research: endorsed by the American Psychiatric Association. *Circulation, 118*(17), 1768–1775. 10.1161/CIRCULATIONAHA.108.190769 10.1161/CIRCULATIONAHA.108.190769 18824640

[97] Chan PS, Jones PG, Arnold SA, Spertus JA (2014). Development and validation of a short version of the Seattle angina questionnaire. Circulation. Cardiovascular Quality and Outcomes.

[98] Porter ME (2010). What is value in health care?. The New England Journal of Medicine.

[99] Porter ME, Lee TH (2016). From volume to value in health care: The work begins. JAMA.

[100] Herlitz J, Hjalmarson R, Karlson BW, Nyberg G (1988). Long-Term morbidity in patients where the initial suspicion of myocardial infarction was not confirmed. Clinical Cardiology.

[101] Schroeder S, Baumbach A, Herdeg C, Oberhoff M, Buchholz O, Kuettner A, Hanke H, Karsch KR (2001). Self-rated health and clinical status after PTCA: Results of a 4-year follow-up in 500 patients. European Journal of Internal Medicine.

