# Supplementary material for: Patient-centered care in Coronary Heart Disease: what do you want to measure? A systematic review of reviews on patient-reported outcome measures
Source: Qual Life Res. 2022 Nov 9;32(5):1405–25. doi: 10.1007/s11136-022-03260-6 (PMC10123044; doi:10.1007/s11136-022-03260-6)
Supplement: Supplementary file 1 — Supplementary file1 (DOCX 443 kb) [file 11136_2022_3260_MOESM1_ESM.docx]

**Supplementary material.**

**Table 4.** Characteristics of PROMs that assess other constructs.

|  | **Publication:**  **Author, year [reference]** | **Instrument** | **Acronym** | **Aim** | **Dimensions** | **Number**  **of items** | **Original language and adaptations** | **Studies published**  **last 10 years** | **Reliability** | **Validity** | **Respon­siveness** |
| --- | --- | --- | --- | --- | --- | --- | --- | --- | --- | --- | --- |
| 1 | Rumbaugh DM,  1964 [67] | Cardiac Adjustment Scale | CAS | “to be used in predicting potential  for return to work” |  | 160 | English (UK) | yes |  | Construct validity |  |
| 2 | Barnason SA,  1992 [68] | Heart Disease Management Questionnaire | HDMQ | “to measure the subject's cognitive knowledge related to heart disease management” | Disease and pathophysiology  Diet  Exercise and activity  Self-care management | 20 | English (USA) | no | Internal consistency | Content validity |  |
| 3 | Barnason SA,  1992 [68] | Cardiac Surgical Patient Perceived Self-efficacy | CSPPSE | “to measure participant perception  of their self-efficacy” | Functional activities  Psychological adjustment  Exercise  Dietary  Self-care management | 20 | English (USA) | no | Internal consistency | Content validity |  |
| 4 | Moser DK,  1995 [70] | Control Attitudes Index/Control Attitudes Scale (original and reviewed) | CAS-R | “to measure the degree to which patients feel they have control related to their cardiac disease” |  | 8 | English (USA)  + 1 language | yes | Internal consistency  Reproducibility | Construct validity |  |
| 5 | Hare DK,  1996 [72] | Cardiac Depression Scale | CDS | “an easily administered self-rating cardiac depression scale, derived from the cardiac patients themselves, for reliable measurement of depression in the range commonly found in this particular population” |  | 26 | English (Australia)  + 8 languages | yes | Internal consistency  Reproducibility | Content validity  Criterion validity  Construct validity | yes |
| 6 | Bennett SJ,  1996 [73] | Cardiac Event Threat Questionnaire | CTQ | “to measure threat related to cardiac events” | Fatigue  General health  Disease-specific symptoms  Work  Family | 32 | English (USA) | yes | Internal consistency  Reproducibility | Construct validity |  |
| 7 | Lerner DJ,  1997 [74] | Angina-related Limitations at Work Questionnaire | ALWQ | “to measure work limitations related to angina” |  | 17 | English (USA) | no | Internal consistency | Content validity  Construct validity |  |
| 8 | Sullivan MD,  1998 [75] | Cardiac self-efficacy scale | CSE | “to elucidate the role that self-efficacy plays in the translation of disease into symptoms and disability in coronary populations” | Symptoms control  Functions maintenance | 13 | English (USA)  + 1 language | yes | Internal consistency | Content validity  Construct validity |  |
| 9 | The ENRICHD Investigators,  2000 [76] | Enhancing Recovery in Coronary Heart Disease social support | ENRICHD-social support | “developed for the ENRICH study by identifying items for structural, instrumental and emotional support previously found to be predictive of mortality individually in cardiovascular patients” |  | 5 or 7 | English (USA)  + 1 language | yes | Internal consistency  Reproducibility | Construct validity |  |
| 10 | Eifert GH,  2000 [77] | Cardiac Anxiety Questionnaire | CAQ | “to measure heart-focused anxiety in persons with and without heart diseases” | Fear  Avoidance  Attention | 18 | English (USA)  + 4 languages | yes | Internal consistency  Reproducibility | Construct validity |  |
| 11 | Barnason SA,  2002 [69] | Barnason Efficacy Expectation Scale | BEES | “to determine the coronary artery bypass grafting patient’s self-efficacy related to risk-related-reduction-aspect of recovery and lifestyle adjustment following surgery” |  | 15 | English (USA)  + 1 language | yes | Internal consistency | Content validity  Construct validity |  |
| 12 | Di Bendetto M.,  2005 [78] | Cardiac Depression Visual Analogue Scale | CDVAS | “a rapid and easy method of assessing  depressed mood in post-acute coronary population” |  | 6 | English (Australia) | yes | Internal consistency  Reproducibility | Content validity  Construct validity |  |
| 13 | Young, Q-R.,  2007 [79] | Screening Tool for Psychological Distress | STOP-D | “to assess psychosocial constructs: depression, anxiety, stress, anger, and social support” |  | 5 | English (Canada) | yes |  | Content validity  Criterion validity |  |
| 14 | Riegel B.,  2007 [71] | Acute Coronary Syndrome Response Index | ACS-RI | “a measure of patient knowledge, attitudes, and beliefs regarding acute coronary syndrome” | Knowledge  Attitude  Belief | 33 | English (Australia, New Zealand, USA)  + 2 languages | yes | Internal consistency | Content validity  Construct validity |  |
| 15 | Abberger B.,  2013 [80–85, 101] | Computer adaptive test for cardiac patients undergoing rehabilitation | RehaCAT  Cardio | “an item bank for the assessment of activities of daily living, anxiety, work capacity and treatment motivation in cardiovascular rehabilitation patients” |  | Item banks | German | yes | Internal consistency | Construct validity | yes ^c^ |
| 16 | Chang CW.,  2014 [86] | Perceptions of coronary heart disease scale | PCS | “to assess individuals’ perceptions of CHD” | Seriousness  Risk | 9 | Chinese | yes | Internal consistency | Content validity  Construct validity |  |
| 17 | Steca P.,  2015 [87] | The Cardiovascular Management Self-efficacy Scale | CMSS | “to assess cardiovascular patients’ beliefs in their ability to manage their disease in terms of some important key areas” | Cardiac risk factors  Adherence to therapy  Symptom recognition | 9 | Italian  + 1 language | yes | Internal consistency  Reproducibility | Content validity  Construct validity |  |
| 18 | Odell A.,  2017 [88] | Expectation Questionnaire^a^ | ExpQ | “to assess patient expectations and perceptions before and after the care process including coronary angiography and any subsequent treatment in individuals with suspected CHD” |  | 11 and 7 | Swedish | yes | Reproducibility | Content validity  Construct validity |  |
| 19 | Dickson VV.,  2017 [89] | Self-Care of Coronary Heart Disease Inventory | SC-CHDI | “to reflect the theoretical constructs of self-care maintenance, management and confidence in stable CHD individuals” | Maintenance  Management  Confidence | 22 | English (USA)  + 4 languages (v2)  and English (USA) + 11 languages (v2.2) | yes | Internal consistency | Content validity  Construct validity |  |
| 20 | Jackson A.,  2020 [90] | Cardiac Distress Inventory^b^ | CDI | “a measure of persistent psychological and emotional distress in cardiac patients” |  | - | English + 5 languages in development (Italian, Hebrew, Arabic, Farsi and Spanish.) | in development |  |  |  |

^a^Instrument developed for the study.

^b^Instrument in development (protocol published)

^c^Different forms for specific domains of the RehaCAT-Cardio item bank have been developed examining construct validity and internal consistency reliability based on Item Response Theory and, in the case of the ACAT-cardio (Anxiety in cardiovascular rehabilitation patients), also responsiveness was tested.

**Supplementary material.**

**Search strategy:** (("ischemic heart disease" OR "coronary heart disease" OR "coronary artery disease" OR angina OR "angina pectoris" OR "myocardial infarction" OR "acute coronary disease" OR "ischemic heart failure" OR "myocardial ischemia" OR "coronary disease" OR "acute coronary syndrome" OR "coronary syndrome") AND (questionnaire OR questionnaires OR instrument OR scale OR index OR “patient-reported outcome”)) AND ("quality of life" OR "perceived health" OR "health status") AND Filters: Review, Systematic Review
